# Proteome Responses to Acute Inhibition of De Novo Sphingolipid Synthesis Suggest Cancer Combination Therapies

**DOI:** 10.3390/cancers18111827

**Published:** 2026-06-02

**Authors:** Thi Thu Trang Luu, Dakai Zhang, Khaggeswar Bheemanapally, Masihuz Zaman, Zhiping Wu, Yang Liu, Xiaoqin Wu, Hyun-Eui Kim, Lei Zheng, Besim Ogretmen, Junmin Peng, Guangwei Du

**Affiliations:** 1 Molecular & Translational Biology Program, MD Anderson Cancer Center UTHealth Graduate School of Biomedical Sciences, Houston, TX 77030, USA; 2 Department of Integrative Biology and Pharmacology, McGovern Medical School, The University of Texas Health Science Center at Houston, Houston, TX 77030, USA; 3 Departments of Structural Biology, St. Jude Children’s Research Hospital, Memphis, TN 38105, USA; 4 Department of Biochemistry and Molecular Biology, Center for Membrane Biology, McGovern Medical School, The University of Texas Health Science Center at Houston, Houston, TX 77030, USA; 5 Department of Biochemistry and Molecular Biology, Medical University of South Carolina, Charleston, SC 29425, USA; 6 Departments of Developmental Neurobiology, St. Jude Children’s Research Hospital, Memphis, TN 38105, USA

**Keywords:** de novo sphingolipid synthesis, myriocin, proteome, cholesterol synthesis, lysosome, cancer, combination therapy

## Abstract

**Background:** Sphingolipids are essential structural and signaling lipids that support membrane integrity and govern cell fate decisions. While the consequences of chronic sphingolipid inhibition have been extensively explored, the immediate cellular responses to acute suppression of sphingolipid synthesis remain poorly defined. **Methods:** We analyzed subcellular proteomic changes following an acute reduction in sphingolipid levels induced by myriocin, an inhibitor of de novo sphingolipid synthesis. We then evaluated the cytotoxicity of co-treatment with myriocin and inhibitors of the altered pathways in cancer cells. **Results:** We found that de novo sphingolipid synthesis is sensitive to myriocin, an inhibitor of serine palmitoyltransferase (SPT), and can be efficiently inhibited within 4 h of treatment. Cells respond to reduced sphingolipid levels by rapidly remodeling their proteome. Mass spectrometry analysis revealed changes in the abundance of hundreds of proteins across the membrane, cytosolic, and nuclear fractions. Gene set enrichment analysis revealed alterations in the proteome across several pathways involved in protein and lipid homeostasis and stress responses, including upregulation of cholesterol homeostasis and lysosome. Co-treatment with myriocin and cholesterol synthesis or lysosomal function inhibitors synergistically reduced cancer cell viability by promoting apoptosis rather than other forms of programmed cell death. **Conclusions:** Together, our work provides insights into how cells rapidly rewire the abundance of certain protein classes in response to reduced sphingolipid levels and identifies signaling and metabolic pathways that can be exploited for therapeutic intervention.

## Simple Summary

Sphingolipids are fats that help cells maintain their structure and regulate survival, but how cells respond immediately when sphingolipid production is blocked remains unclear. In this study, we show that treating human cervical cancer cells with myriocin is sufficient to suppress sphingolipid synthesis within 4 h, triggering widespread, rapid changes in protein levels across multiple cellular compartments. These changes affect pathways involved in stress responses and lipid metabolism, including increased expression of proteins linked to cholesterol regulation and lysosomal function. Importantly, combining sphingolipid inhibition with drugs that block cholesterol production or lysosome function strongly increases cancer cell death, revealing how knowledge of proteomic changes following short-term lipid disruption uncovers new vulnerabilities in cancer cells.

## 1. Introduction

Maintaining lipid homeostasis is critical to cellular structure, function, and viability [1,2]. By balancing the synthesis and turnover of different lipids, cells maintain cellular integrity and performance across physiological and stress conditions, ensuring organismal health [1,2]. Accordingly, a systematic analysis of cellular responses to the disruption of specific lipid metabolic pathways is necessary to uncover how cells respond to external and internal alterations, and to design strategies that either improve homeostasis to enhance the functions of normal cells or disrupt it to remove diseased cells.

Sphingolipids are a class of membrane lipids built on a sphingoid base [3,4]. Sphingolipids are not only critical components of cell membranes but also central regulators of signaling. Mutations in sphingolipid metabolic enzymes are associated with severe genetic disorders, and the dysregulation of sphingolipid levels has been implicated in many human diseases, including cancer, neurodegeneration, immune disorders, and metabolic disease [3,4,5,6]. Therefore, there is considerable interest in targeting these lipids and the signaling pathways that regulate their metabolism. Genetic knockout or knockdown and pharmacological approaches have been employed to investigate the molecular and cellular responses that occur when sphingolipid metabolism is inhibited [7,8,9,10]. These studies have generated insights into how cells respond to changes in sphingolipid levels; however, they cannot distinguish whether these responses are direct or compensatory because the analyses were performed after manipulating sphingolipid metabolism for a few days or longer. Therefore, it is critical to understand the signaling and metabolic pathways that control the early cellular changes in response to myriocin treatment, as these may represent direct responses that counteract the reduction in sphingolipid levels to sustain cell proliferation and viability. Knowledge gained from such studies would not only provide insights into the regulation of sphingolipid metabolism but also identify actionable targets for therapies.

In the current study, we analyzed subcellular proteomic changes in HeLa cells in which sphingolipid levels were acutely reduced by inhibiting de novo sphingolipid synthesis with myriocin, a potent and highly specific inhibitor of serine palmitoyltransferase (SPT), the first and rate-limiting enzyme in the de novo sphingolipid synthesis pathway [3,7]. Through pathway analysis, we found that the proteins in a few biochemical or metabolic pathways, including cholesterol homeostasis and lysosome, are altered as early as 4 h after myriocin treatment. Co-treatment of myriocin and lovastatin (an inhibitor of de novo cholesterol synthesis) synergistically reduced the viability of several cancer cell lines. Similarly, combined treatment with myriocin and bafilomycin A1 (a lysosomal inhibitor) also synergistically reduced HeLa cell viability. Our work thus reveals early proteomic responses to acute inhibition of sphingolipid synthesis and suggests effective targets for cancer combination therapy.

## 2. Materials and Methods

### 2.1. General Reagents and Antibodies

Small-molecule inhibitors myriocin (MilliporeSigma, Burlington, MA, USA, catalog no. M1177), bafilomycin A1 (MedChemExpress, Monmouth Junction, NJ, USA, catalog no. HY100558), torin 1 (Cayman Chemical, Ann Arbor, MI, USA, catalog no. 10997), and trametinib (MedChemExpress, catalog no. HY-10999) were used in this study. Primary antibodies included anti-LAMP1 (Developmental Studies Hybridoma Bank, Iowa City, IA, USA, catalog no. AB_528127), hFAB™ Rhodamine anti-actin (Bio-Rad, Hercules, CA, USA, catalog no. 12004163), anti-tubulin (MilliporeSigma, catalog no. T5168), anti-HSD17B7 (Proteintech, Rosemont, IL, USA, catalog no. 14854-1-AP), anti-ATP6V0D2 (Proteintech, catalog no. 33364-1-AP), anti-cleaved caspase-3 (Cell signaling, Danvers, MA, USA, catalog no. 9661S), anti-pMLKL (Cell signaling, catalog no. 91689S), anti-MLKL (MilliporeSigma, catalog no. MABC604), and anti-GSDMD (Abclonal, Woburn, MA, USA, catalog no. A20197). Secondary antibodies included HRP-conjugated goat anti-rat IgG (Abclonal, catalog no. AS028), Goat anti-mouse IgG conjugated with Alexa Fluor™ 680 (ThermoFisher Scientific, Waltham, MA, USA, catalog no. A-21058), Goat anti-Rabbit IgG conjugated with Alexa Fluor™ 680 (ThermoFisher Scientific, catalog no. A-21076), and Goat anti-Rabbit IgG conjugated with DyLight™ 800 4X PEG (ThermoFisher Scientific catalog no. SA5-35571).

### 2.2. Cell Culture

HeLa (ATCC, CCL-2), HCT116 (ATCC, CCL-247), MCF7 (ATCC, HTB-22), and MDA-MB-231 (ATCC, HTB-26) cells were cultured at 37 °C and 5% CO_2_ in Dulbecco’s Modified Eagle Medium (DMEM) (HyClone^TM^, Logan, UT, USA, catalog no. SH30243.01) supplemented with 10% fetal bovine serum (FBS) (MilliporeSigma, F0926). Cells were verified by short tandem repeat (STR) profiling and routinely confirmed to be free of mycoplasma contamination. Inhibition of de novo sphingolipid synthesis was performed by incubating cells with 1.5 μM myriocin in DMEM containing 10% lipid-depleted FBS for the indicated times, as reported before [9], and the effectiveness of myriocin was confirmed by mass spectrometry. To prepare lipid-depleted FBS, 500 mL of FBS was incubated with 25 g of lipid-removal adsorbent resin (LRA) (MilliporeSigma, 13358-U) under constant rotation at 4 °C overnight. Resin particles were then removed by two sequential centrifugation steps: first at 2000× *g* for 5 min to eliminate bulk resin, and second at 27,000× *g* for 30 min to remove fine particles. The resulting FBS was adjusted to pH 7.4 and filter-sterilized through a 0.2 µm polyethersulfone (PES) membrane.

### 2.3. Crude Cellular Fractionation

The crude cellular fractionation was performed as described previously [11]. Briefly, 3.5 × 10^6^ HeLa cells were seeded on 150 mm dishes and treated the following day with either DMSO or 1.5 μM myriocin in DMEM supplemented with 10% lipid-free FBS for 4 h. After treatment, cells were washed with buffer 1 (20 mM HEPES–KOH, pH 7.4) and harvested in 2 mL of buffer 2 (20 mM HEPES–KOH, pH 7.4, 10 mM KCl), followed by centrifugation at 500× *g* for 2 min at 4 °C. Cell pellets were resuspended in 2 mL of fractionation buffer (0.25 M sucrose, 20 mM HEPES–KOH, pH 7.4, 1 mM EDTA, 10 mM KCl, supplemented with protease inhibitor cocktail) and homogenized using an Isobiotec cell homogenizer (Heidelberg, Germany) with 16-μm clearance beads. Eight and six strokes were required to break DMSO- and myriocin-treated cells, respectively, to achieve the same level of cell membrane disruption, as reduced sphingolipid levels by myriocin treatment decreased membrane rigidity. The homogenates were centrifuged at 800× *g* for 10 min to separate the nuclear pellet and post-nuclear supernatant. The supernatant was transferred to a new 1.5 mL microtube and subjected to ultracentrifugation at 100,000× *g* for 30 min, yielding the cytosolic supernatant and membrane pellet. Nuclear and membrane pellets were resuspended in 2 mL PBS supplemented with protease inhibitor cocktail. All fractions were prepared in 40 μL of 1× SDS loading buffer (3% sucrose, 56.4 mM Tris, 2.7% SDS, 2 mM EDTA, 1% 2-mercaptoethanol, 0.01% bromophenol blue), and stored at −80 °C for later mass spectrometry protein analysis or Western blotting analysis.

### 2.4. Protein Analyses by Mass Spectrometry

Proteins in the membrane, cytosol, and nucleus were run on a 10% SDS-PAGE gel for 7 min to remove salts and other low-molecular-weight contaminants. Gel lanes were excised and reduced with 10 mM dithiothreitol (DTT) at 55 °C for 20 min, followed by alkylation with 55 mM iodoacetamide at room temperature in the dark for 30 min. Proteins were then digested in-gel with trypsin at an enzyme-to-protein ratio of 1:25 (*w*/*w*) overnight. Resulting peptides were analyzed by data-independent (DIA) LC/MS/MS. Briefly, peptides were separated on a reversed-phase column (75 µm × 20 cm, 1.7 µm C18 resin; CoAnn Technologies (Richland, WA, USA) coupled to an Orbitrap Exploris 480 mass spectrometer (Thermo Fisher Scientific) via a Dionex UltiMate 3000 nanoLC system. Peptides were eluted at 65 °C using a 12–36% buffer B gradient over 40 min (buffer A: 0.1% formic acid in water with 3% DMSO; buffer B: 0.1% formic acid in 67% acetonitrile with 3% DMSO) at a flow rate of 0.25 µL/min. The mass spectrometer operated in positive-ion mode using DIA, comprising one full MS scan followed by 30 MS/MS scans. MS1 spectra were acquired at a resolution of 60,000 with an AGC target of 3 × 10^6^, scan range of *m*/*z* 500–1100, and maximum injection time of 25 ms. MS2 spectra were acquired at a resolution of 30,000 with a fixed first mass of *m*/*z* 120, an AGC target of 1 × 10^6^, a maximum injection time of 22 ms, and a 20 *m*/*z* isolation window.

Raw data were analyzed using diaNN (version 1.8.2) against a human protein database containing 83,955 entries. Carbamidomethylation of cysteine was set as a fixed modification, while methionine oxidation was included as a variable modification. Peptide- and protein-level false discovery rates (FDRs) were controlled at 1%. Differentially expressed proteins and peptides were identified using the limma R package (version 3.62.2) of the online JUMPshiny server [12,13]. Statistical significance was determined based on *p*-values and log_2_(FC) thresholds. Proteins were considered differentially abundant if they met both a statistical significance threshold (two-tailed *p* < 0.05) and an effect-size threshold defined as two standard deviations (2×SD) from the fitted Gaussian distribution of protein-level variability.

### 2.5. Western Blotting

Western blotting was performed as described before [14,15]. Total whole cells or different subcellular fractions were harvested in 1× PBS supplemented with phosphatase inhibitors (1 mM Na_3_VO_4_, 5 mM NaF, 3 mM β-glycerophosphate, and 4 mM sodium tartrate) and a protease inhibitor cocktail (Roche Diagnostics, Indianapolis, IN, USA, catalog no. 004-693-116-001). Thirty micrograms of protein, denatured in 1× SDS loading buffer by boiling, were separated by SDS–PAGE and transferred onto PVDF membranes (Cytiva, Marlborough, MA, USA, catalog no. 10600022) using a semi-dry transfer system. Membranes were blocked in 0.5% casein or AzureSpectra Fluorescent Blot Blocking Buffer (Azure Biosystems, Dublin, CA, USA, catalog no. S1044) and incubated with primary antibodies diluted in the same buffer supplemented with Tween-20. After washing, membranes were incubated with fluorescently labeled secondary antibodies. Protein signals were detected using a Sapphire Biomolecular Imager (Azure Biosystems, Dublin, CA, USA) and band intensities were quantified with ImageJ (version 1.53e). For pMLKL and total MLKL detection, pMLKL was first detected and stripped, followed by reprobing the same membrane with an anti- MLKL antibody. Cleaved caspase-3 and its loading control β-actin were detected on separate membranes using the same protein lysates. The results presented were representative of at least 3 experiments.

### 2.6. Viability Assays

A total of 1.8 × 10^4^ HeLa cells and 2.5 × 10^4^ HCT116, MCF7, and MDA-MB-231 cells were seeded into 24-well plates. The following day, cells in lipid-free medium were treated with a series of myriocin concentrations (0, 4, 40, 400, and 4000 nM) in the absence or presence of a fixed concentration of the lovastatin, bafilomycin A1, torin 1, or trametinib, as indicated in the figure legend. Cells were then fixed and stained with 0.1% crystal violet (MilliporeSigma, catalog no. C0775) in methanol. After washing and air-drying, the dye was eluted with methanol. The optical density was measured at 570 nm using a Synergy 2 plate reader (BioTek, Winooski, VT, USA). Cell viability in each condition was normalized to that of DMSO-treated control cells and was performed in three independent experiments. The combination effect was evaluated using the Bliss independence model [16], based on the mean cell viability values from replicate measurements of single-agent and combination treatments performed under the same experimental conditions. Bliss scores were calculated as $E_{1,2}-(E_{1}+E_{2}-E_{1}E_{2})$, where $E_{1}$ and $E_{2}$ represent the inhibitory effects of each drug alone and $E_{1,2}$ represents the observed effect of the drug combination. Bliss scores were interpreted relative to the theoretical Bliss value: positive values indicate synergy, near-zero values indicate additivity, and negative values indicate antagonism.

### 2.7. Sphingolipid Measurement

4.0 × 10^5^ HeLa cells per well were seeded into 6 cm dishes and grown for 48 h. Cells were then treated with either DMSO or 1.5 μM myriocin for 4 h in medium supplemented with 10% lipid-free FBS. After treatment, cells were harvested by trypsinization, resuspended in 1000 μL PBS, and 50 μL of the suspension was removed for protein quantification. The remaining suspension was centrifuged to collect cell pellets, which were stored at −80 °C. Three independent cultures were analyzed for each strain, and relative levels of each lipid were normalized to total protein levels determined by the BSA method. Briefly, cells were extracted in 2.0 mL of isopropanol:water:ethyl acetate (30:10:60) (by volume), sonicated for 30 s, vortexed, and centrifuged for 5 min at 3000× *g*. The upper organic phase was dried under nitrogen. Lipids were reconstituted in 150 µL of 1 mM ammonium formate in methanol containing 0.2% formic acid. Sphingolipids were analyzed by HPLC-MS/MS using a Vanquish uHPLC system coupled to a Quantis Plus triple quadrupole mass spectrometer (Thermo Fisher Scientific) equipped with an ESI probe operating in the multiple reaction monitoring (MRM) positive ion mode at the Medical University of South Carolina Lipidomics Shared Resource, as previously described [17,18]. Briefly, chromatographic separations are obtained by gradient elution on a C8 column using a mobile phase containing ammonium formate and formic acid in water and methanol. Quantitative analyses of sphingolipids are based on eight-point calibration curves generated for each target analyte. The synthetic standards, along with a set of internal standards, are spiked into an artificial matrix and then subjected to the same extraction procedure as the biological samples. These extracted standards are then analyzed with the samples by the HPLC-MS/MS. Peaks for the target analytes and internal standards are recorded and processed using the instrument’s software Xcalibur 4.7. Plotting the analyte/internal standard peak area ratios against analyte concentration yields sphingolipid-specific calibration curves. Any sphingolipids for which no standards are available are quantified using the calibration curve of their closest counterpart.

### 2.8. Pathway Enrichment Analysis

Gene Set Enrichment Analysis (GSEA) analysis was performed at http://www.broad.mit.edu/gsea (accessed on 20 February 2026) [19] to identify Hallmark [20] and KEGG [21] pathways of interest. Significantly enriched pathways were determined in GSEA by a false discovery rate (FDR) < 0.2 and an absolute normalization enrichment score (|NES|) > 1.4.

### 2.9. Statistics

All data are shown as means ± SD from three independent experiments. For two-group comparisons, an unpaired two-tailed Student’s *t*-test was used. For experiments involving multiple conditions or dose–response analyses, two-way ANOVA was used followed by Šídák’s multiple comparisons test. A *p*-value < 0.05 was considered statistically significant. *, *p* < 0.05, **; *p* < 0.01; ***, *p* <0.001, and ****, *p* < 0.0001.

## 3. Results

### 3.1. Sphingolipid Levels Are Sensitive to Acute Inhibition of De Novo Sphingolipid Synthesis by Myriocin

**Figure 1:**
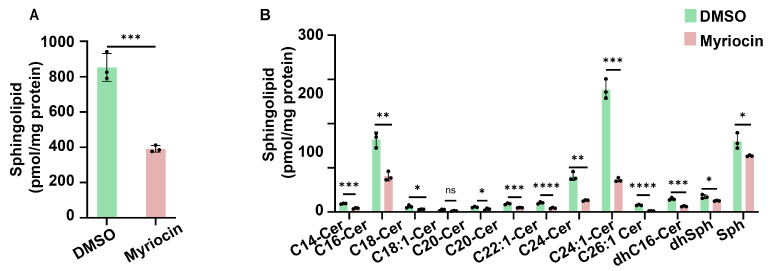
Sphingolipid levels are sensitive to short-term myriocin treatment in HeLa cells. (**A**) Total sphingolipid and (**B**) individual sphingolipid species in HeLa cells treated with either DMSO or 1.5 µM myriocin for 4 h (*n* = 3). Statistical analysis was performed by an unpaired two-tailed Student’s *t*-test. Each point represents the mean ± standard deviation of a triplicate experiment (ns: not significant, * *p* < 0.05; ** *p* < 0.01; *** *p* < 0.001; **** *p* < 0.0001). Cer, ceramide; dhC16-Cer, C16 dihydroceramide; dhSph, sphinganine; Sph, sphingosine.

Cells can quickly respond to changes in lipid levels by coordinately adjusting the metabolic activities of other macromolecules and activating feedback regulatory pathways to maintain lipid homeostasis [3]. To understand the acute and early proteome responses to changes in sphingolipid levels, we treated HeLa cells with myriocin for 4 h, then collected cells to measure the levels of the intermediate products of de novo sphingolipid synthesis, including ceramides with various lengths of fatty acid acyl chain, sphinganine, and sphingosine using mass spectrometry. The data showed that this condition resulted in approximately a 54% reduction in total levels of these sphingolipids (Figure 1A, Supplemental Table S1). The inhibition of ceramide levels appears to be independent of the length and saturation of the fatty acid acyl chains of the ceramides, although those with long fatty acid acyl chains (C20 to C26) show greater reductions (Figure 1B, Supplemental Table S1). This result indicates that cells are sensitive to myriocin treatment and that acute inhibition of de novo sphingolipid synthesis can cause a drastic reduction in sphingolipid levels, which we hypothesize can trigger adaptive cellular responses.

### 3.2. Acute Inhibition of De Novo Sphingolipid Synthesis Triggers Rapid Proteome Remodeling

**Figure 2:**
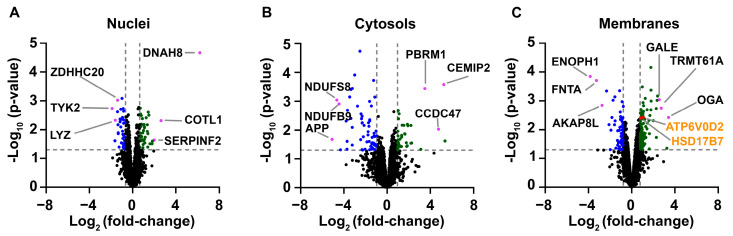
Volcano plots highlighting differentially abundant proteins between control and myriocin -treated HeLa cells. Volcano plots display differentially abundant proteins within each subcellular fraction: (**A**) nuclear, (**B**) cytosolic, and (**C**) membrane, using two standard deviations (2×SD) and a *p*-value < 0.05. Each dot represents an individual protein. Upregulated and downregulated differentially abundant proteins are shown in green and blue, respectively. Black dots represent non-differentially abundant proteins. Dashed horizontal and vertical lines indicate *p*-value and fold-change significance thresholds, respectively. The x-axis represents log_2_ fold-change in protein abundance, and the y-axis represents −log_10_ *p*-values. A few proteins in each fraction and the two proteins involved in cholesterol homeostasis and lysosome pathways, ATP6V0D2 and HSD17B7, are highlighted.

In addition to changes in protein levels, the regulation of some signaling or metabolic pathways, such as de novo cholesterol synthesis, is also involved in changes in subcellular localization, e.g., translocation of SREBP2 from cytoplasmic organelles to the nucleus [22,23]. To better understand cellular responses to reduced sphingolipid levels, we performed a crude subcellular fractionation in HeLa cells treated with or without myriocin. Cells in a hypotonic buffer were broken down by mechanical force and separated into three fractions by differential centrifugation. While the simple differential spins in this procedure cannot fully separate the intended subcellular fractions, they are expected to provide useful information for spatial changes in most proteins. The intended fractionations were confirmed by immunoblotting, as indicated by Histone H3, α-tubulin, and LAMP1 as markers for the nuclei, cytosols, and membranes, respectively (Supplementary Figure S1). The proteins in each fraction were subjected to mass spectrometry for identification. The identified proteins were then analyzed and presented with the online JUMPshiny server [13]. In total, 5627 proteins were identified in the nuclear fraction, 4630 in the cytosolic fraction, and 5598 in the membrane fraction (Supplemental Table S2). Even with a short treatment of myriocin for four hours, a few hundreds of differentially abundant proteins could be determined using two standard deviations (2×SD) and a *p*-value < 0.05 in the three subcellular fractions (Figure 2): in the nuclear fraction, 40 and 44 proteins were decreased and increased, respectively; in the cytosolic fraction, 62 and 37 proteins were decreased and increased, respectively; in the membrane fraction, 53 and 139 proteins were decreased and increased, respectively.

### 3.3. Acute Inhibition of De Novo Sphingolipid Synthesis Leads to Proteomic Changes in Pathways Involved in Protein and Lipid Homeostasis and Stress Responses

**Figure 3:**
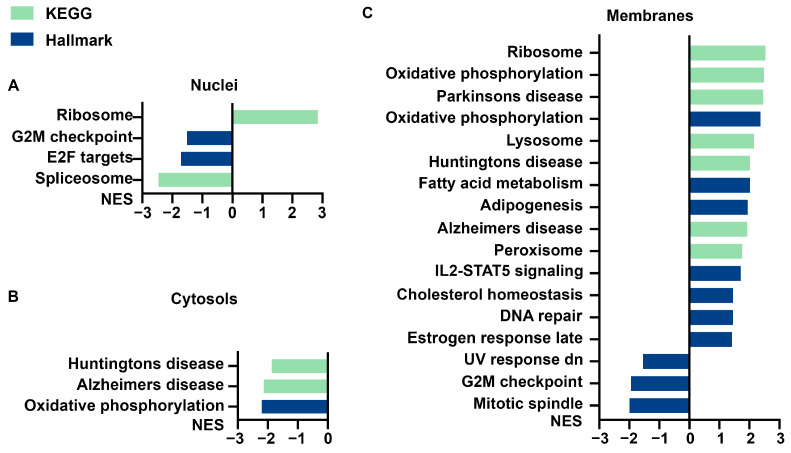
Global pathway enrichment analysis of proteins following acute sphingolipid depletion. Hallmark and KEGG pathways from Gene Set Enrichment Analysis (GSEA) of proteins in each cellular fraction: (**A**) nuclear, (**B**) cytosolic, and (**C**) membrane, with or without acute sphingolipid inhibition, were ordered by normalized enrichment score (NES). Significantly enriched pathways were defined as those with a false discovery rate (FDR) < 0.2 and an absolute NES > 1.4.

To understand how acute myriocin treatment alters signaling and metabolic pathways, we performed Gene Set Enrichment Analysis (GSEA) [19] across all quantified proteins in each cellular fraction using either Hallmark [20] or Kyoto Encyclopedia of Genes and Genomes (KEGG) terms [21], with a false discovery rate (FDR) < 0.2 and an absolute normalized enrichment score (|NES|) > 1.4 being considered as significantly enriched (Figure 3). The most altered terms in both Hallmark and KEGG analyses are in the membrane fraction. In the Hallmark analysis, the nuclear fraction shows negative enrichment of G2M checkpoint and E2F targets. In the cytosolic fraction, the oxidative phosphorylation is negatively enriched. In the membrane fraction, positively enriched pathways include oxidative phosphorylation, fatty acid metabolism, adipogenesis, IL2-STAT5 signaling, cholesterol homeostasis, DNA repair, and estrogen response late, whereas negatively enriched pathways include UV response down, G2M checkpoint, and mitotic spindle. Complementary patterns are revealed across different subcellular compartments in the KEGG analysis. The nuclear fraction shows positive and negative enrichment for ribosome and spliceosome, respectively. The cytosolic fraction shows negative enrichment for Huntington’s disease and Alzheimer’s disease, and no positive enrichment. In the membrane fraction, the positively enriched KEGG pathways include ribosome, oxidative phosphorylation, Parkinson’s disease, lysosome, Huntington’s disease, Alzheimer’s disease, and peroxisome, whereas no negatively enriched pathway is identified.

In broad terms, most of the Hallmark and KEGG pathways are involved in cellular functions related to protein and lipid homeostasis and stress responses. The increase in ribosomes in both nuclear (expected to contain some membranes in our crude subcellular fractionations) and membrane fractions suggests that an increase in the translation of transmembrane proteins on the rough endoplasmic reticulum, such as those involved in cholesterol homeostasis and fatty acid metabolism, is an early response to sphingolipid deficiency.

### 3.4. Co-Treatment of Myriocin and the Inhibitor of De Novo Cholesterol Synthesis or Lysosomal Function Causes Synergistic Cytotoxicity in HeLa Cells

**Figure 4:**
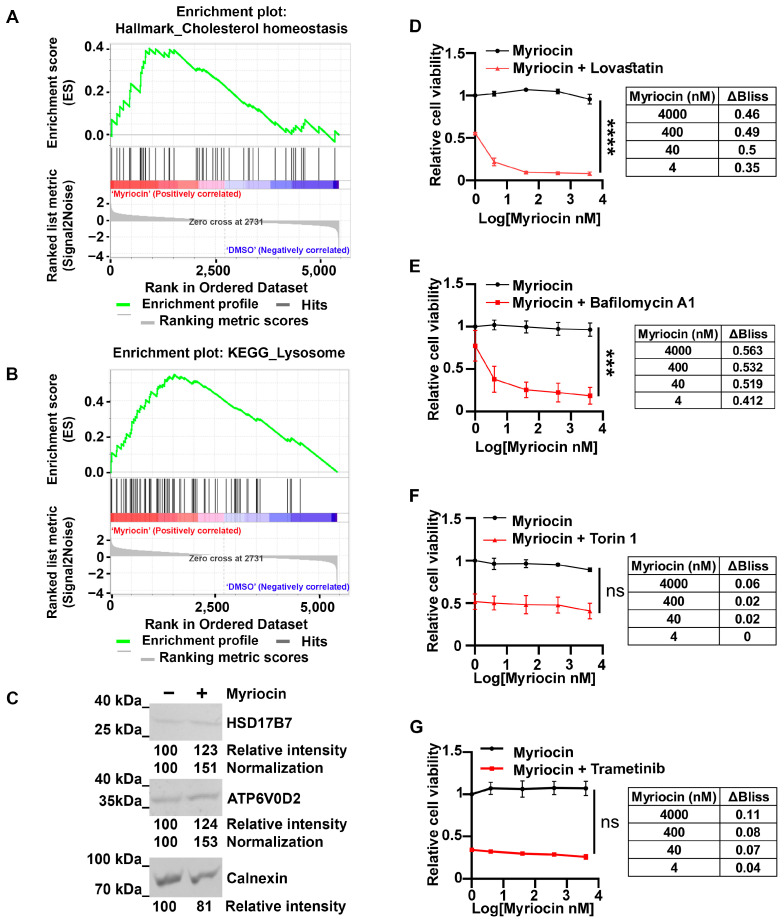
Co-treatment with myriocin and lovastatin, or with myriocin and bafilomycin A1, resulted in synergistic inhibition of HeLa cell viability. GSEA result of the cholesterol homeostasis (**A**) and lysosome (**B**) pathways. The x-axis shows the proteins, ranked from most enriched to most downregulated between the control and treatment groups. The y-axis shows the normalized enrichment score (NES). (**C**) Western blot analysis of HSD17B7 and ATP6V0D2 in the membrane fraction. HeLa cells were treated with DMSO or 1.5 µM myriocin for 4 h, followed by isolation of the crude membrane fraction. Band intensities were quantified using ImageJ and normalized to Calnexin. Relative protein levels were calculated as fold changes relative to the control. (**D**–**G**) Synergistic analyses of the combined treatment of myriocin and selected inhibitors. Myriocin dose–dependent cytotoxicity of HeLa cells in the absence (control) or presence of (**D**) lovastatin (5 µM, 5 days), (**E**) bafilomycin A1 (400 nM, 5 days), (**F**) torin 1 (0.01 µM, 3 days), and (**G**) trametinib (0.1 µM, 5 days). The first data points on the left represent either no treatment, lovastatin alone, bafilomycin A1 alone, Torin 1 alone, or Trametinib alone. Based on the cell viability results, Bliss scores were calculated to assess the combinatorial effects of myriocin with each selected inhibitor. A score greater than 0.1 indicates a synergistic effect. Each point represents the mean ± standard deviation from three independent measurements. Statistical analysis was performed by a 2-way ANOVA followed by Šídák’s multiple comparisons test to compare single- and combination-treatment conditions. *** *p* < 0.001; **** *p* < 0.0001; ns: not significant. The uncropped Western blotting images can be found in Supplementary Materials.

The changes in selected signaling and metabolic pathways induced by acute myriocin treatment suggest that they mediate early, adaptive responses critical to cell proliferation and viability under sphingolipid deficiency. We reasoned that co-targeting some of these upregulated pathways would sensitize cells to de novo sphingolipid synthesis inhibition, thereby providing guidance for the treatment of certain human diseases, such as cancer. To test this idea, we selected two pathways that are positively enriched by myriocin treatment, and that can be inhibited by available drugs known to be well tolerated, de novo cholesterol synthesis and lysosome (Figure 4A,B), for further investigation. Among these proteins, the cholesterol biosynthesis enzyme HSD17B7 and the lysosomal vacuolar H^+^-ATPase (V-ATPase) subunit ATP6V0D2 are two of the top upregulated proteins by myriocin treatment, increasing by 80% and 110%, respectively, in mass spectrometry data (Supplemental Table S2). Western blotting analyses confirmed that their levels were upregulated, although the increases from the controls to the treatments were smaller, i.e., 51% for HSD17B7 and 53% for ATP6V0D2 (Figure 4C), which may reflect differences in detection method between mass spectrometry and Western blotting. Cell viability assay showed that myriocin treatment alone had minimal effect on cell viability at various concentrations (0–4 μM) (Figure 4D). While lovastatin and Bafilomycin A1 treatment alone reduced the number of cells compared with the no-treatment control, there was no sign of cell death (Figure 4D,E, Supplementary Figure S2). In contrast, co-treatment with myriocin and either lovastatin, a cholesterol synthesis inhibitor, or bafilomycin A1, a lysosomal V-ATPase inhibitor, produced synergistic effects on cytotoxicity across the tested dose ranges, as determined by the Bliss score [16] (Figure 4D,E). As controls, we also tested the outcomes of combined inhibition of de novo sphingolipid synthesis and of pathways not enriched by myriocin treatment, such as mTOR and MAPK. Although treatment with the mTOR inhibitor torin 1 or with the MAPK signaling inhibitor trametinib alone reduced cell viability, their combination with myriocin showed minimal additional toxicity in HeLa cells (Figure 4F,G).

### 3.5. Combined Treatment of Myriocin and Lovastatin Synergistically Promotes Cell Death in HCT116, MDA-MB-231, and MCF7 Cancer Cells

**Figure 5:**
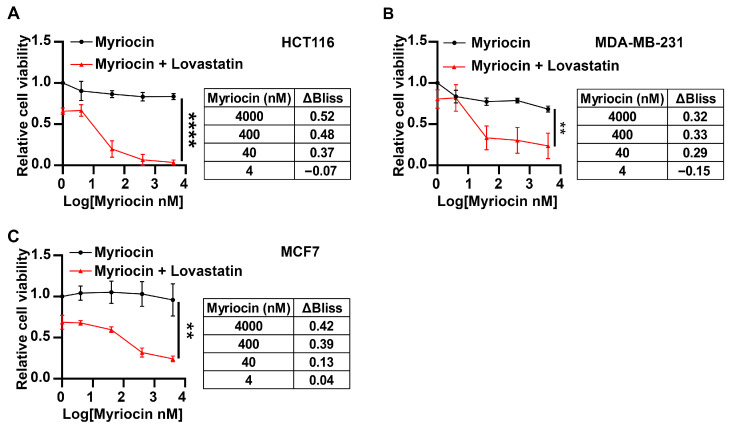
Myriocin and lovastatin exhibit synergistic inhibition of the viability of HCT116, MDA-MB-231, and MCF7 cells. Myriocin dose–response curves showing cell viability in the absence (control) or presence of lovastatin in (**A**) HCT116 (2 µM), (**B**) MDA-MB-231 (0.2 µM), and (**C**) MCF7 (2 µM) cells, for 3 days. The first data points on the left represent either no treatment or lovastatin alone. Based on the cell viability results, Bliss scores were calculated to assess the combinatorial effects of Myriocin with each selected inhibitor. A score greater than 0.1 indicates a synergistic effect. Each point represents the mean ± standard deviation from three independent measurements (some error bars are smaller than the symbols). Statistical analysis was performed using a two-way ANOVA followed by Šídák’s multiple comparisons test to compare single- and combination-treatment conditions. ** *p* < 0.01; **** *p* < 0.0001.

We then examine whether our findings from HeLa cells, derived from the uterine cervix, can be extended to other cancer cell lines of different tissue origins, such as HCT116 (colon), MDA-MB-231 (breast), and MCF7 (breast), using the combined treatment of myriocin and lovastatin as a proof of principle. As with HeLa cells, myriocin treatment alone showed only a cytostatic effect in all 3 cell lines, and lovastatin treatment alone showed a moderate cytotoxicity at the tested concentrations in HCT116 and MCF7 cells. However, the combined treatment with myriocin and lovastatin showed synergistic effects in reducing the viability of all three cancer cell lines (Figure 5), supporting the notion that knowledge gained from HeLa cells can be applied to other cancers.

### 3.6. Combined Treatment of Lovastatin and Myriocin Leads to Activation of Apoptosis Marker

**Figure 6:**
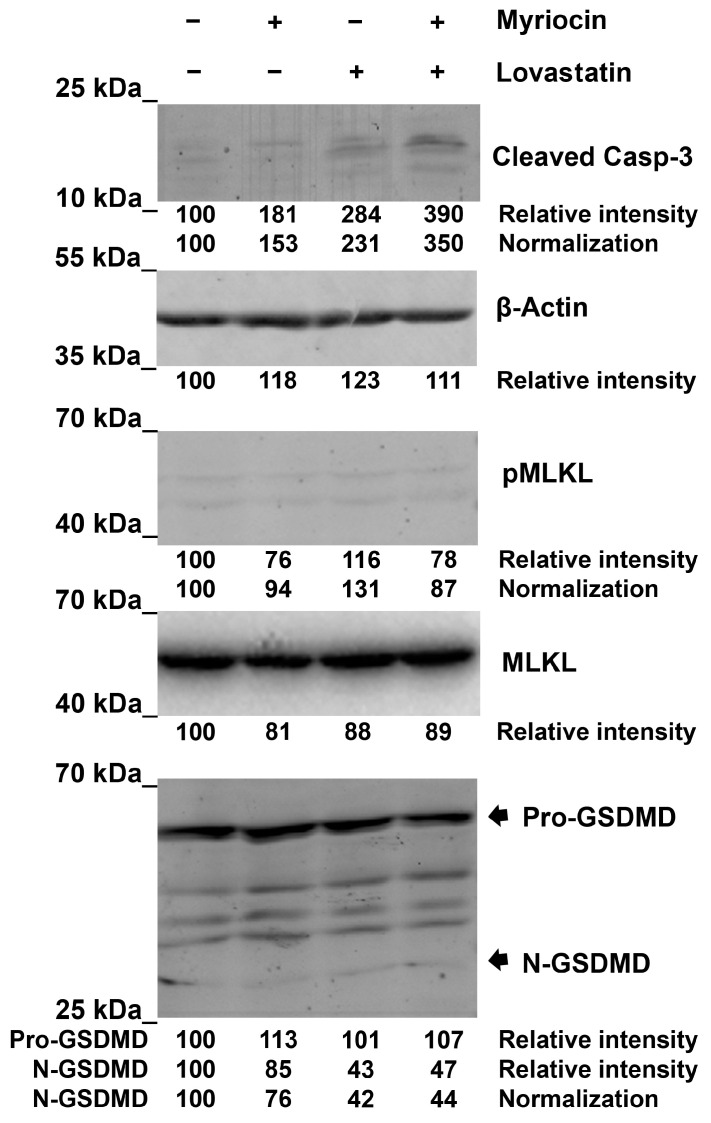
Combined myriocin and lovastatin treatment induces apoptotic cell death in HeLa cells. HeLa cells were treated with DMSO (control), myriocin (1.5 µM), lovastatin (5 µM), or both. Whole-cell lysates were analyzed by Western blot for cleaved caspase-3, pMLKL, MLKL, N-GSDMD, GSDMD, and β-Actin. β-Actin, MLKL, and pro-GSDMD served as loading controls for cleaved Casp-3, pMLKL, and cleaved N-GSDMD, respectively. The uncropped Western blotting images can be found in Supplementary Materials.

To understand the molecular basis of cell death induced by the combined treatments, we performed Western blotting using lysates from cells treated with vehicles, myriocin, lovastatin, or both myriocin and lovastatin, using the markers for different types of cell death [24,25], e.g., cleaved caspase 3 (apoptosis), phospho-MLKL (Ser358) (pMLKL) (necroptosis), and cleaved N-terminal fragment of GSDMD (N-GSDMD) (pyroptosis). In line with cell viability, individual drug treatment had no or some effect; however, combined treatment with myriocin and lovastatin induced a significant increase in the apoptotic marker cleaved caspase-3 (Figure 6). In contrast, the same treatments showed only basal activation of the necroptosis and pyroptosis markers, pMLKL and N-GSDMD, and the combined treatment didn’t increase their levels (Figure 6).

## 4. Discussion

While cellular responses to de novo sphingolipid synthesis have been investigated in great detail at the single-gene and global levels in the past [9,10,26], our current study provides new insights. We focus on early responses to acute inhibition of de novo sphingolipid synthesis rather than on cellular responses after long-term inhibition, as reported in previous studies, to minimize complications caused by secondary or compensatory mechanisms. By combining short-term myriocin treatment with subcellular proteomics, we show that inhibition of de novo sphingolipid synthesis leads to rapid remodeling of several signaling/metabolic pathways related to protein and lipid homeostasis and stress responses, including lysosome and cholesterol homeostasis (Figure 3). Another difference in our study is that we investigate proteome changes, whereas previous studies primarily focused on the consequences of chronic inhibition of sphingolipid synthesis on transcriptional and functional changes. For example, the transcriptome was profiled in myriocin-treated HT22 cells, which showed that downregulated genes are mainly related to cell proliferation and extracellular regulation, whereas upregulated gene signatures are responses to hypoxia, transcription regulation, cAMP signaling, membrane, mitochondria, and MAPK activity [10]. In another study using CRISPRi library screening to identify myriocin-co-dependent genes, myriocin-sensitive gene sets include endoplasmic reticulum-Golgi transport, cholesterol metabolism, membrane trafficking, and ether lipid synthesis, whereas myriocin-resistant gene sets include mRNA regulation and post-translational modification [9]. The changes in pathways/processes identified in our study partially overlap with the CRISPRi screening, such as cholesterol metabolism and mRNA regulation, but minimally overlap with the transcriptome analysis, suggesting that the acute proteome responses can provide good indications for the dependence of proteins required for cell survival/proliferation under de novo sphingolipid synthesis inhibition. We also identified processes missed in the CRISPRi screening but crucial for survival/proliferation during sphingolipid inhibition (Figure 3), such as the lysosomal pathway, underscoring the value of complementary approaches for identifying functionally important proteins.

Cells have developed elegant feedback regulatory mechanisms to maintain cholesterol homeostasis by controlling the stability of rate-limiting enzymes, such as 3-hydroxy-3-methylglutaryl coenzyme A reductase (HMGCR) and squalene monooxygenase, or the activity of the master regulator, such as SREBP2, through proteolysis or changes in subcellular localization [22,23]. One of our initial goals of the current study was to investigate whether a similar mode of posttranslational regulation exists in the de novo sphingolipid synthesis pathway. However, we didn’t identify changes in either protein levels or subcellular localization of enzymes/regulators involved in sphingolipid metabolism after 4 h of myriocin treatment. In contrast, we observe upregulation of enzymes in the cholesterol homeostasis pathway (Figure 3C and Figure 4A,C, Supplemental Table S2). This finding is consistent with the previous finding that the dependence of cholesterol synthesis genes in myriocin-treated cells [9]. There might be four reasons why we didn’t identify a feedback regulation similar to those in cholesterol synthesis. First, cholesterol upregulation may serve as an acute compensatory mechanism to counteract decreased sphingolipid levels and maintain the biophysical properties of cell membranes, since both lipids are critical to the formation of liquid-ordered membrane microdomains [27]. Indeed, previous studies have demonstrated the stabilization of membrane nanodomains by hydrogen-bond interactions between sphingolipids and cholesterol [27] and the interplay between cholesterol and sphingolipid synthesis [9,28]. Second, the cellular response to changes in sphingolipid levels is relatively slower, so no significant change in protein levels was detectable after 4 h of myriocin treatment in our condition. Third, the feedback regulation of sphingolipid synthesis may primarily occur through mechanisms other than changes in protein abundance and localization, such as post-translational modifications of enzyme activity or protein–protein interactions. For example, the only direct regulators of sphingolipid synthesis identified so far are the ORM proteins (ORMDL in mammalian cells), which bind to SPT and mediate negative feedback regulation of sphingolipid synthesis [3,29]. Fourth, ceramides and sphingosine can be generated by the hydrolysis of membrane sphingomyelin and glycosphingolipids in lysosomes [3,4], which may slow the responses of the relevant enzymes when the de novo sphingolipid synthesis pathway is inhibited. The importance of lysosomes in sphingolipid homeostasis is supported by our finding that cells are sensitive to combined inhibition of de novo sphingolipid synthesis and lysosomal activity. To investigate whether additional regulatory mechanisms beyond protein abundance exist to regulate sphingolipid levels, it is necessary to examine not only protein abundance but also post-translational modifications and the protein interactome at different time points during myriocin treatment.

The cellular responses identified in our study can provide guidance for the treatment of human diseases involving ceramides. Ceramides are generally known to contribute to the pathology of metabolic and cardiovascular diseases, and inhibition of de novo ceramide production has been proposed for their treatment [4,6]. The role of ceramides in cancer is paradoxical. While ceramides are generally considered proapoptotic, increases in some ceramide species and certain ceramide-synthesizing enzymes are correlated with poor outcomes [4,5]. Accordingly, inhibiting certain enzymes at the early steps of the de novo sphingolipid synthesis pathway demonstrated anticancer activity [4,30,31]. A recent study also suggests a promising cancer-targeting strategy by inducing the accumulation of the toxic metabolic intermediate 3-ketodihydrosphingosine through inhibition of 3-ketodihydrosphingosine reductase (KDSR), which lies downstream of SPT [32]. We have demonstrated that while inhibiting SPT with myriocin alone produced only a cytostatic effect, its combination with lovastatin and bafilomycin A1 resulted in synergistic killing in several cancer cell lines of different tissue origins and in HeLa cells, respectively. Since these combination strategies are based on responses in cells after short-term exposure to myriocin, it is more likely that cancer cells can be eliminated before later adaptive survival mechanisms are activated. Taken together, these studies highlight the distinct cellular effects of inhibiting different de novo sphingolipid-synthesizing enzymes in cancer cells, supporting the idea that inhibiting de novo sphingolipid synthesis, alone or in combination with targeting cellular stress responses induced by sphingolipid deficiency, represents a promising approach for treating cancer.

## 5. Conclusions

In conclusion, our work demonstrates that inhibiting de novo sphingolipid synthesis leads to rapid changes in the levels of specific proteins in certain signaling and metabolic pathways. These early responses reveal the vulnerability of cancer cells to de novo sphingolipid synthesis inhibitors, supporting the idea that knowledge of early proteome changes can guide the development of improved cancer combination therapy.

## Data Availability

The datasets used during the current study are included in the Supplementary Materials. Additional information is available from the corresponding author upon request.

## Supplementary Materials

The following supporting information can be downloaded at https://www.mdpi.com/article/10.3390/cancers18111827/s1: Figure S1. Validation of the quality of cell fractionation in myriocin-treated HeLa cells. Figure S2. Determination of the sensitivity of HeLa cells to different bafilomycin A1 concentrations. Table S1. Quantitative analysis of sphingolipid species in control and myriocin-treated samples. Table S2. Mass spectrometry analysis of proteins altered by myriocin treatment in different cellular fractions. File S1: The uncropped original Western blotting images. The mass spectrometry proteomics data have been deposited to the ProteomeXchange Consortium via the PRIDE [33] partner repository with the dataset identifier PXD079016.
