# Supplementary material for: Proteome Responses to Acute Inhibition of De Novo Sphingolipid Synthesis Suggest Cancer Combination Therapies
Source: Cancers (Basel). 2026 Jun 2;18(11):1827. doi: 10.3390/cancers18111827 (PMC13256006; doi:10.3390/cancers18111827)
Supplement: Supplementary file 1 [file cancers-18-01827-s001.zip › Supplemental Figures.pdf]

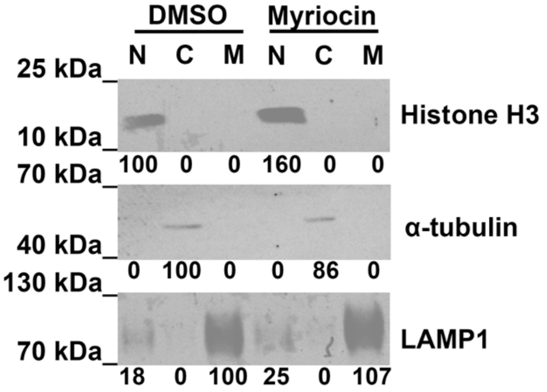

Supplemental Figure S1. Validation of the quality of cell fractionation in myriocin-treated HeLa cells. HeLa cells were treated with either DMSO or 1.5 μM myriocin for 4 hours. Cells in each condition underwent crude fractionation to separate into different cellular fractions. A Western Blot was conducted to evaluate the quality of each fraction. LAMP1, α-tubulin, and Histone H3 were used as markers for membranes (M: Membrane), cytosol (C), and nucleus (N), respectively.

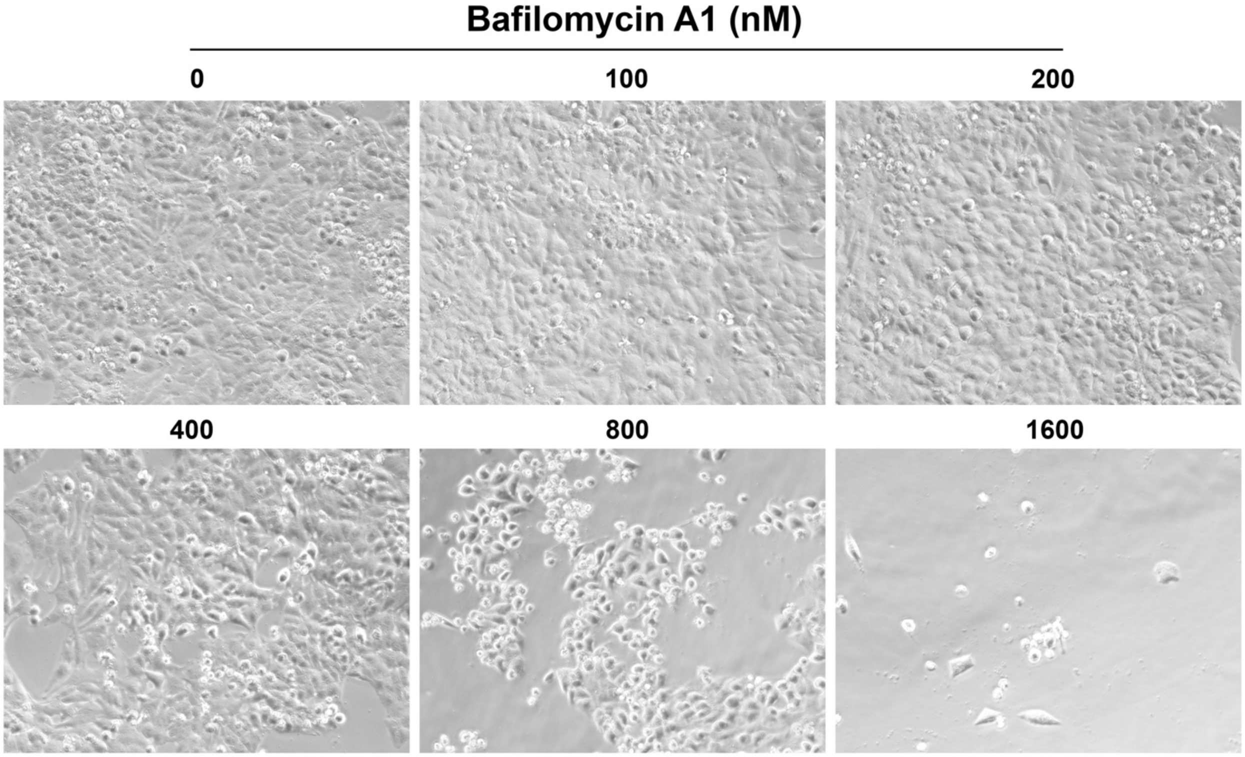

Supplemental Figure S2. Determination of the sensitivity of HeLa cells to different bafilomycin A1 concentrations. HeLa cells cultured in DMEM medium containing 10 % lipid-depleted FBS were treated with either DMSO or bafilomycin A1 at the indicated concentrations for 5 days. Representative images were captured using an EVOS M5000 imaging system (Invitrogen, Thermo Fisher Scientific) with a 20× objective lens. 400 nM bafilomycin A1 was selected for subsequent experiments, combining it with different concentrations of myriocin.
