# Supplementary figures and images for: Proteome Responses to Acute Inhibition of De Novo Sphingolipid Synthesis Suggest Cancer Combination Therapies
Source: Cancers (Basel). 2026 Jun 2;18(11):1827. doi: 10.3390/cancers18111827 (PMC13256006; doi:10.3390/cancers18111827)

Figure 4

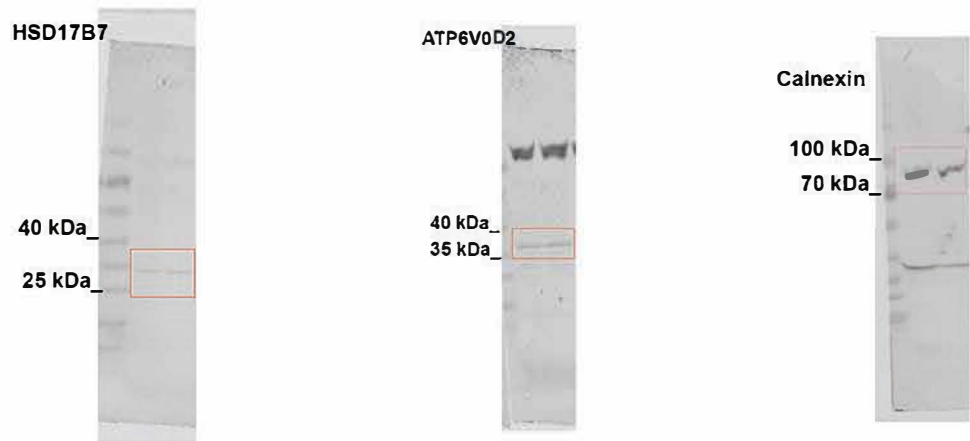

Figure 6

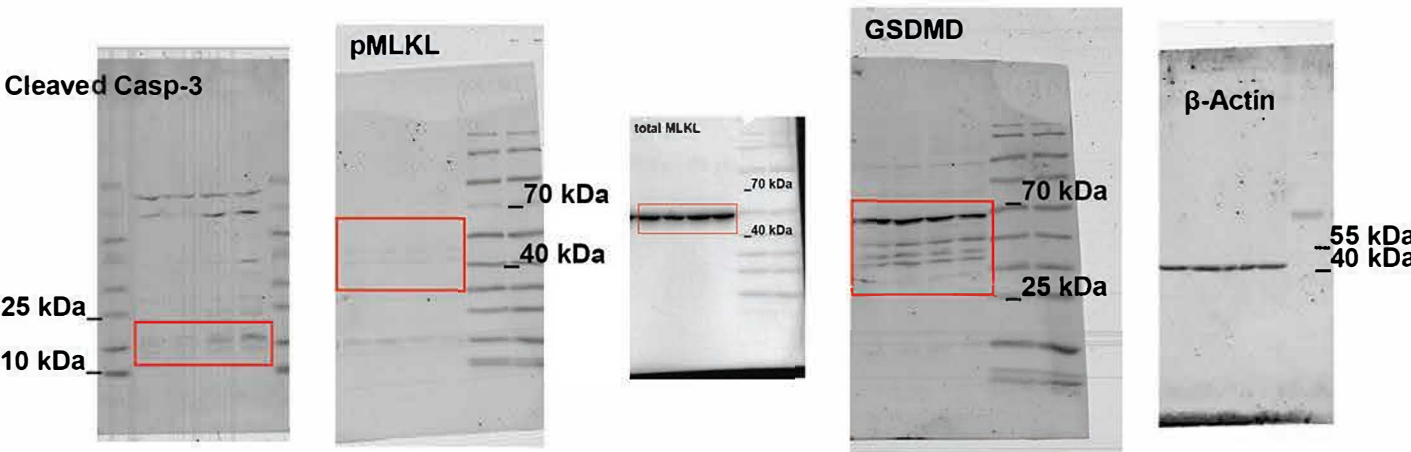

Supplemental figure S1

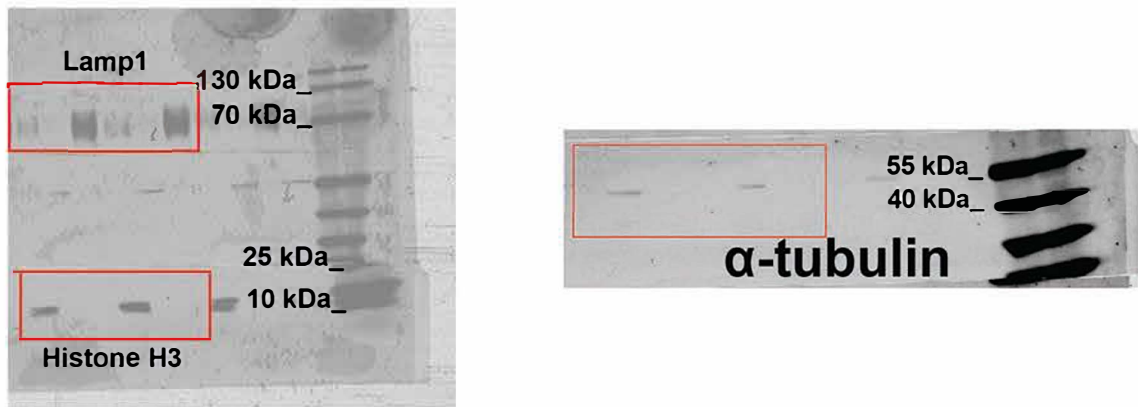

Supplement: Supplementary file 1 [file cancers-18-01827-s001.zip › File S1. The uncropped original Western blotting images.pdf]
